# Developmental Milieu Influences a Gene's Role in Tumor Formation

**DOI:** 10.1371/journal.pbio.0020375

**Published:** 2004-09-28

Whether a person inherits a defective gene or acquires genetic damage by chance, two types of genes typically play a role in transforming a healthy cell into a cancer cell. Oncogenes and tumor suppressor genes are normally involved in cell growth, development, and cell differentiation. Both functions can be appropriated to ill effect by mutations. Single mutations in these genes rarely cause cancer on their own, but they predispose cells to additional insults that precipitate malignant transformation.

Susceptibility to cancer depends, among other things, on age. Though cancer in children is rare, the most common childhood cancers strike the hematopoietic system (leukemia), nervous system, and skeletal muscle system, while solid tumors of the lung, breast, prostate, and colon are more common in adults. This age differential suggests that an oncogene's ability to cause cancer in a particular cell type might depend on that cell's developmental stage. (A cell's gene expression profile differs with type and age; breast cells express different genes than liver cells, and immature cells express different genes than fully differentiated cells.) In a new study, Dean Felsher and colleagues show that age matters: activating oncogenes at different developmental time points in mouse liver cells produces different results.

Typically, once a cell is transformed, it stays in its “differentiative” state, that is, it stays in whatever developmental stage it was in when it became a tumor cell. But in a previous study, Felsher and colleagues found that turning off oncogenes in tumor cells allowed them to differentiate; these mature cells did not resume tumorigenesis after the oncogenes were reactivated. In this study, Felsher and colleagues show that the ability of the *MYC* oncogene to initiate liver cancer (hepatocellular carcinoma) in a transgenic mouse model varies with the age of the mouse.

**Figure:**
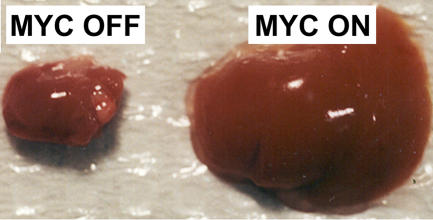
Developmental consequences of MYC overexpression

To study the consequences of *MYC* overexpression in the liver cells of embryonic, neonatal, and adult mice, the authors used a biotech trick (called the Tet system) that controls gene expression dose and timing with a drug. The system relies on the interplay of two elements: a gene (in this case, *MYC*) fused to a regulatory enhancer, and a transcription factor that binds to the enhancer and activates the gene. Administering a tetracycline-like drug (in this case, doxycycline) prevents the transcriptional activation of the gene.

Overexpressing the *MYC* oncogene in mice during embryonic development or at birth occasioned their demise fairly quickly (ten days and eight weeks after birth, respectively). In contrast, overexpression of *MYC* in adult mice resulted in tumorigenesis only after a long latency period. When the authors evaluated the cellular effects of *MYC* overexpression, they found that hepatocytes from neonatal transgenic mice showed evidence of increased proliferation (replicated DNA content) compared to normal hepatocytes, while transgenic adult hepatocytes showed increased cell and nuclear growth (some nuclei had as many as twelve genome copies instead of two) without dividing. Since these adult cells eventually developed into tumors, some clearly acquired the ability to divide, which the authors show is facilitated, among other events, by the loss of the p53 tumor suppressor.

Altogether these results suggest that whether oncogene activation can support tumor growth depends on the age of the host, which in turn suggests the role of genetically distinct pathways in young and adult mice. The consequences of *MYC* activation, Felsher and colleagues conclude, depend on the cell's developmental program, which determines whether a cell can grow and divide, or simply grow. In adult hepatocytes—which are normally quiescent—*MYC* requires additional genetic events to induce cell division and tumorigenesis; in immature hepatocytes—which are already committed to a program of cellular proliferation—*MYC* activation alone is sufficient. The next step will be to identify the epigenetic developmental factors, both internal and external, that lead to tumor formation, and how to prevent it.

